# Long COVID in Young Patients: Impact on Lung Volume Evaluated Using Multidetector CT

**DOI:** 10.3390/tomography9040101

**Published:** 2023-06-30

**Authors:** Davide Bellini, Paola Capodiferro, Simone Vicini, Marco Rengo, Iacopo Carbone

**Affiliations:** 1 Department of Medical-Surgical Sciences and Biotechnologies, Sapienza University of Rome–Academic Diagnostic Imaging Division, I.C.O.T. Hospital, Via Franco Faggiana, 1668, 04100 Latina, Italy; bellini.davide@uniroma1.it (D.B.); marco.rengo@uniroma1.it (M.R.); 2 Department of Radiological Sciences, Oncology and Pathology, Sapienza University of Rome–Academic Diagnostic Imaging Division, I.C.O.T. Hospital, Via Franco Faggiana, 1668, 04100 Latina, Italy; paola.capodiferro@uniroma1.it (P.C.); iacopo.carbone@uniroma1.it (I.C.)

**Keywords:** Long COVID, lung volume, quantitative chest CT, SARS-CoV-2, coronavirus disease

## Abstract

Purpose: To evaluate using quantitative analysis on chest CT images a possible lung volume reduction in Long COVID patients who complain mild respiratory symptoms, with chest CT negative for inflammatory findings. Materials and Methods: CT images of patients from 18 to 40 years old who underwent chest CT scan at our institution were analyzed retrospectively, using AwServer Thoracic VCAR software for a quantitative study. Exclusion criteria were inflammatory findings at CT, previous lung surgery, lung cancer, and breath artifacts that invalidate the quality of images. Patients were divided into two groups: in the first one (“post-COVID”) were patients who had previous SARS-CoV-2 infection, confirmed by an RT-PCR, who underwent chest CT from 3 to 6 months after their negativization for long COVID symptoms; in the control group (“non-COVID”), were enrolled patients who underwent a chest CT scan from January 2018 to December 2019, before the spread of COVID in Italy. Results: Our final population included 154 TC, 77 post-COVID patients (mean age 33 ± 6) and 77 non-COVID patients (mean age 33 ± 4.9). Non statistical significative differences were obtained between groups in terms of age, sex, and other characteristics that affect total lung capacity such as obesity, thoracic malformations, and smoking habit. Mean values of the total lung volume (TV), right-lung volume (RV), and left-lung volume (LV) in the post-COVID group compared with non-COVID group were, respectively: 5.25 ± 0.25 L vs. 5.72 ± 0.26 L (*p* = 0.01); 2.76 ± 0.14 L vs. 3 ± 0.14 L (*p* = 0.01); 2.48 ± 0.12 L vs. 2.72 ± 0.12 L (*p* = 0.01). Conclusion: In patients with symptoms suggesting Long COVID and negative chest CT macroscopic findings, quantitative volume analysis demonstrated a mean value of reduction in lung volume of 10% compared to patients of the same age who never had COVID. A chest CT negative for inflammatory findings may induce clinicians to attribute Long COVID mild respiratory symptoms to anxiety, especially in young patients. Our study brings us beyond appearances and beyond the classic radiological signs, introducing a quantitative evaluation of lung volumes in these patients. It is hard to establish to what extent this finding may contribute to Long COVID symptoms, but this is another step to gain a wider knowledge of the potential long-term effects caused by this new virus.

## 1. Introduction

Long COVID syndrome gained widespread recognition among social support groups and later in scientific and medical communities. This condition is still poorly understood as it affects patients previously infected by severe acute respiratory syndrome coronavirus 2 (SARS-CoV-2) at all levels of disease severity, even younger adults, children, and those who had never been hospitalized. While the precise definition of Long COVID may be lacking, the most common symptoms reported in many studies are fatigue and dyspnea that last for months after acute infection. Other persistent symptoms may include cognitive and mental impairments, chest and joint pains, palpitations, myalgia, smell and taste dysfunctions, cough, headache, gastrointestinal and cardiac issues [1].

Despite acute COVID being milder in children and young people, it should not be assumed that such patients at low risk of life-threatening acute infection do not suffer the long-term consequences of SARS-CoV-2. It is plausible that they may be at higher risk of suffering from Long COVID, given that much is still unknown in young people about the immunological susceptibility and the underlying biology of Long COVID [2].

Many studies demonstrated that patients in early convalescence phase after acute infection often show impaired diffusing-capacity and lower respiratory muscle strength. Notwithstanding the utility of clinical tests to demonstrate the lung impairment in Long COVID patients (e.g., Diffusion Lung CO, Spirometry, and 6 Minute Walking test), imaging has a pivotal role [3].

Chest CT is the best radiological test in the diagnosis and follow-up of patients with COVID [4,5,6,7]. Numerous studies have documented several radiological findings in the acute course of infection, from mild to severe [8,9,10,11]. Moreover, in more than half of the COVID patients, fibrotic-like changes, ground-glass opacities (GGOs), consolidations, pulmonary interstitial thickening, and crazy paving pattern may last in the early convalescence phase (30 days after acute infection) [3], as also demonstrated by chest digital tomosynthesis (DTS) [12] and repeated lung ultrasound [13].

Recently published papers [14,15] found that approximately 94% of hospitalized patients have persistent lung parenchymal findings on their discharge CT scans. On the other hand, the majority of the evidence suggests that lung alterations resolve with no adverse sequelae within 3 months and within 3 weeks in 53% of patients with mild COVID. For these reasons, most of the patients with symptoms suggesting long COVID do not show any particular macroscopic findings at chest CT, especially young people with mild respiratory symptoms.

Data from previous coronavirus infections (i.e., severe acute respiratory syndrome and Middle East respiratory syndrome) suggest that there may be substantial fibrotic long-term consequences [16,17,18]. However, little is known about the long-term lung changes after COVID infection. The evaluation of lung volumes could be considered a surrogate to identify this condition.

With this in mind, the aim of our study was to evaluate a potential lung volume reduction in Long COVID patients, demonstrating no lung inflammatory or fibrotic abnormalities at chest CT imaging, as compared to sex- and age-matched controls, using a quantitative CT analysis, possibly suggesting subtle fibrotic changes not detectable at standard imaging.

## 2. Material and Methods

This single-center, retrospective, observational, case–control study was approved by the Institutional Review Board of Sapienza University of Rome (approval code: 88SPR0722).

The requirement for written informed consent was waived. Reporting was performed according to the Strengthening the Reporting of Observational Studies in Epidemiology (STROBE) recommendations [19].

### 2.1. Setting and Participants

We searched the Picture Archiving and Communication System (PACS) (syngo.plaza, Siemens Healthineers, Erlange, Germany) for consecutive outpatients, aged 18 to 40 years, who had prior SARS-CoV-2 infection confirmed by Real-Time Reverse Transcriptase-Polymerase Chain Reaction (RT-PCR), and who were referred for chest CT from 3 to 6 months after negativization (from December 2021 to July 2022), as requested by the treating physician, due to complaints of mild respiratory symptoms suggesting Long COVID (at least one of cough, dyspnea, chest pain, or fatigue). Only subjects demonstrating normal lung parenchyma on chest CT images were included. Exclusion criteria included inflammatory and/or fibrotic lung changes on CT, known chronic obstructive pulmonary disease (COPD), previous lung surgery, evidence of lung masses or gross consolidations, and non-diagnostic chest CT because of respiratory motion.

Following the same inclusion and exclusion criteria, control participants were identified as outpatients who were required to undergo chest CT due to the same mild respiratory symptoms from January 2018 to December 2019, before the spread of COVID-19 pandemic in Italy, and showed normal lung parenchyma on CT images. One control patient was sampled for each case with prior SARS-CoV-2 infection, matched with respect to sex and age (±2 years), using the PACS at our Institution.

For included patients, age, sex, and symptoms at the time of the CT examination (cough, dyspnea, chest pain, or fatigue), as well as known factors potentially affecting lung capacity (including obesity, thoracic malformations, and smoking habit) [20,21,22], were extracted from the electronic medical record.

### 2.2. CT Image Acquisition Technique

CT examinations were performed using CT scanner GE LightSpeed (GE HealthCare, Chicago, IL, USA) (from January 2018 to December 2019) and CT scanner Siemens Somatom Force Dual Source Dual Energy (Siemens Healthineers, Erlangen, Germany) (from December 2021 to July 2022). In both cases, we used the same scan time (3–5 s) and delay from the start of breath hold message and the beginning of the acquisition (6 s). Scans were performed during a single breath-hold, after a recorded message inviting to take a maximum inspiration.

CT Acquisition parameters were 120 or 100 kV according to patients’ body weight, 100 mAs with automatic current tube modulation, and iterative reconstruction technique (ASIR) using CT scanner GE LightSpeed; Dual Source Dual Energy CT acquisition parameters were: tube current 80 and 150 kV, automatic exposure control (CareDose4D; Siemens Healthineers, Erlangen, Germany) with a quality reference setting of 250 milliampere-second (mAs). In both cases, CT scans were performed with 1 mm reconstruction slice thickness.

### 2.3. Image Analysis and CT Lung Volume Measurements

For evaluation purposes, all chest CT examinations were retrieved from the PACS, anonymized, and displayed on a diagnostic workstation enabling manipulation and processing of CT images (Advantage Workstation version 4.5, GE HealthCare, Chicago, IL, USA). The order of the examinations was randomized.

Each CT examination was retrospectively interpreted in consensus by two board-certified consultant radiologists with more than 5 years of experience in thoracic imaging. Both radiologists were blinded to clinical-anamnestic data and had no specific information regarding prior SARS-CoV-2 infection status in the study population.

All images were evaluated using Thoracic VCAR (Siemens Healthineers, Erlangen, Germany), an Artificial intelligence (AI) based software analysis package for the Advantage Workstation (AW) platform, CT Scanner, Cloud, or PACS stations, designed for the analysis and processing of volumetric CT chest data. It provides quantitative information to aid in the assessment of thoracic diseases.

The primary features calculated by the software are lung and lobe segmentation to obtain threshold-based volume measurements; bronchial tree segmentation and trackas well determine wall thickness measurements; lung maps based on HU values to help the radiologist in determining the location and extent of disease across both lungs a well as each lobe.

**Figure 1:**
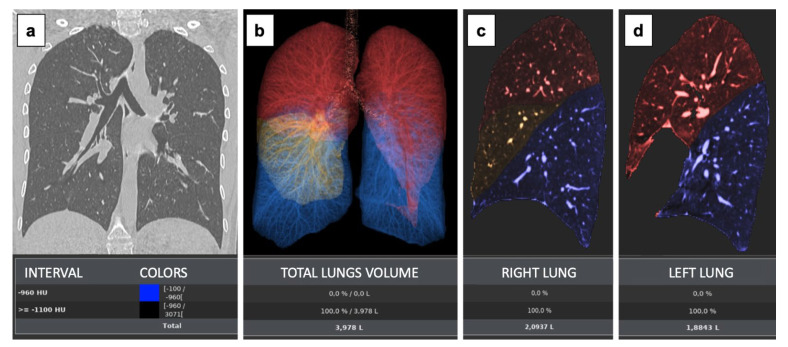
The Parenchyma Analysis Review step offers a tool to measure the volume between different HU ranges. This protocol provides a measurement/workflow tool that can aid for the assessment of lung diseases. User selectable thresholds can be set to define possible abnormal ranges for the lung parenchyma The regions thus found are visually displayed in the images with different colours (blue colour is for emphysema [not present]; red represent the upper lobes; and yellow the middle lobe) (**a**). Their measurements are captured and presented in statistics panel including measurements of total lung volume (**b**) and individual lung volumes (**c**,**d**).

The Parenchyma Analysis Review step offers a tool to measure the volume between different H.U. ranges. Right lung volume (RV) left lung volume (LV) and the total lung volume (TV) have been collected for all patients. Details of the image analysis are reported in Figure 1.

### 2.4. Statistical Analysis

Findings regarding demographic and clinical characteristics (including age, sex, symptoms, and factors affecting lung capacity), and CT lung volume measurements were summarized for the study sample and compared in terms of post-COVID cases and non-COVID controls.

Categorical variables (sex, symptoms, and factors affecting lung capacity) were reported as frequencies with percentages, and continuous variables (age, and CT lung volume measurements) were expressed as means with standard deviations (SD), with corresponding confidence intervals (CIs), as needed. Categorical variables were compared between groups using the Chi-Squared test, used to compare the distribution of a categorical variable in a sample with the distribution of a categorical variable in another sample. Continuous variables were compared between groups using the Mann–Whitney U test which is used to compare differences between two independent groups when the dependent variable is either ordinal or continuous, but not normally distributed.

For all comparisons, statistical significance was assumed to be *p* < 0.05. All statistical analyses were performed by using a commercially available statistical software package (MedCalc Statistical Software version 20.218, MedCalc Software Ltd., Acacialaan 22, Ostend, Belgium).

## 3. Results

### 3.1. Participants

The initial search of cases identified 117 patients, of which 40 were excluded for the following reasons: inflammatory and/or fibrotic lung changes (n = 20), known chronic obstructive pulmonary disease (COPD) (n = 5), previous lung surgery (n = 3), evidence of lung masses or gross consolidations (n = 5), and non-diagnostic chest CT because of respiratory motion (n = 7). Eventually, 77 cases with prior SARS-CoV-2 infection with no radiologically detectable lung parenchymal abnormalities at chest CT were enrolled (hereafter, post-COVID patients).

This patient’s group (mean age 33 ± 6 years) included 39 women (51%) and 38 men (49%), 8 obese patients (10%), 2 patients with thoracic malformations (1.5%) and 17 smokers (22%).

**Figure 2:**
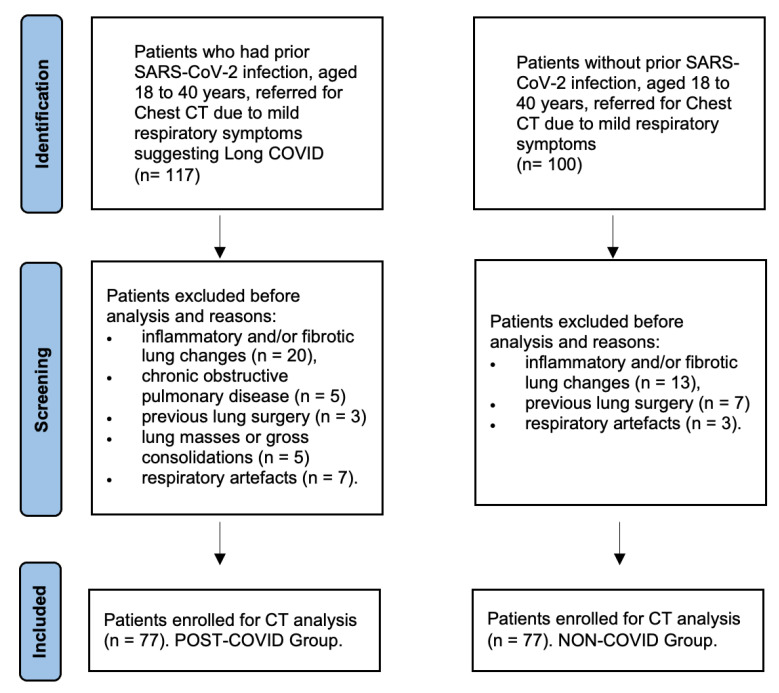
Study flow diagram of patient recruitment. The initial search of cases identified 117 patients with prior SARS-CoV-2 infection who underwent chest CT due to Long COVID symptoms, of which 40 were excluded for the reasons presented in the diagram. Eventually, 77 cases with prior SARS-CoV-2 infection with no radiologically detectable lung parenchymal abnormalities at chest CT were enrolled (Post-COVID Group). As for control group, 100 patients were initially identified, of which 20 were excluded for the reasons presented in the diagram. Eventually, 77 sex- and age-matched patients were selected as controls (Non-COVID Group).

As for control group, 100 patients were initially identified. Twenty were excluded due to inflammatory and/or fibrotic lung changes (n = 13), previous lung surgery (n = 7), or respiratory artefacts (n = 3). Eventually, 77 patients were selected (mean age 33 ± 4.9 years, including 40 women (52%) and 37 men (48%), 9 obese (11%), 3 with thoracic malformations (4%), and 22 smokers (28%)). Study flow diagram of patient selection is presented in Figure 2.

Study population features did not show any statistically significant differences about age, sex, symptoms, and other characteristics that can potentially affect total lung capacity such as obesity, thoracic malformations (e.g., scoliosis), and smoking habit (Table 1).

### 3.2. Chest CT Lung Volume Measurements

Chest CT quantitative analysis results demonstrated a mean RV significantly lower in post-COVID patients [mean RV = 2.76 L (95% CI 2.16–3.36)] than in non-COVID patients [mean RV = 3 L (95% CI 2.43–3.57)] (*p* = 0.017). The mean LV was significantly lower in the post-COVID group [mean LV = 2.48 L (95% CI 1.94–3.02)] as compared to the non-COVID group [mean LV = 2.72 L (95% CI 2.18–3.26)] (*p* = 0.010). Additionally, the mean TV was significantly lower in post-COVID patients ([mean TV = 5.25 L (95% CI 4.13–6.37)] than in non-COVID patients [mean TV = 5.72 L (95% CI 4.62–6.82)] (*p* = 0.012). Table 2 summarizes the comparison in CT lung volume measurements between the two groups.

**Figure 3:**
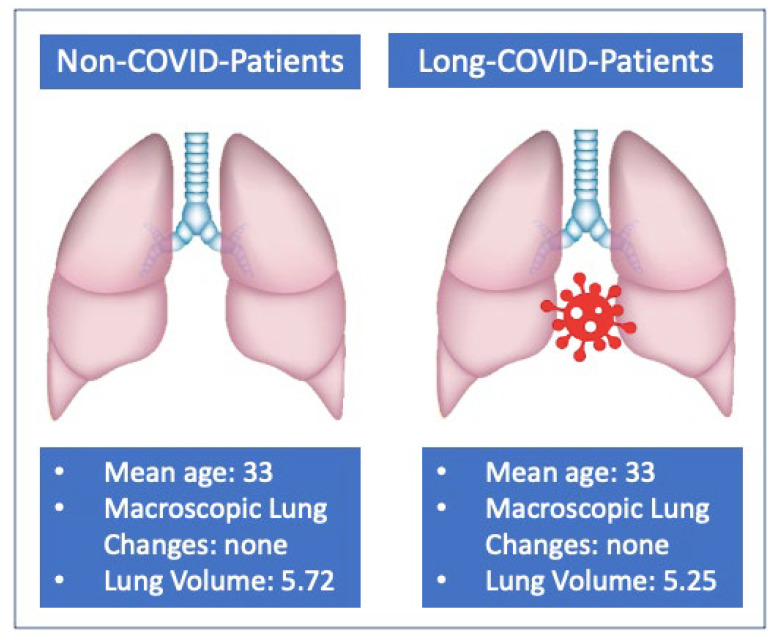
Summary of the main findings regarding lung volume comparison.

In the post-COVID group a mean reduction of 240 mL for both RV and LV, as well as a mean reduction of 480 mL for the TV, were noted as compared to the control group (Figure 3).

## 4. Discussion

CT represents a widely used approach for the assessment of lung sequalae caused by COVID. Our study demonstrates that quantitative chest CT analysis may be an important tool in the management of patients suffering from Long COVID, suggesting persistent microscopic lung changes which may not be present in patients without prior SARS-CoV-2 infection experiencing the same symptoms, introducing new parameters that could enrich the clinical assessment of these patients. Considering young patients who experienced COVID in the preceding 6 months and complained of mild respiratory symptoms, whose chest CT was negative for inflammatory findings, we actually found a significant reduction in lung volume compared to control group.

The post-COVID group showed a mean TV of 5.25 L (95% CI 4.13–6.37 L) compared to the non-COVID group (5.72 L, 95% CI 4.62–6.82 L). Accordingly, negative post-COVID chest CT scans concealed an interesting element, that is a reduction in lung volume of 10% (*p* = 0.01) compared to patients of the same age who never had SARS-CoV-2 infection.

Lung abnormalities on chest CT scans showed the greatest severity approximately 10 days after initial onset of symptoms [9] and a six-month follow-up study revealed that about two third of patients who survived severe coronavirus disease chest CT were negative for fibrotic-like findings; the remaining one third that showed fibrotic-like changes were associated with an older age (>50 years), acute respiratory distress syndrome, longer hospital stays, tachycardia, noninvasive mechanical ventilation, and higher initial chest CT score [23]. Two recent studies also accurately evaluated interstitial lung abnormalities in patients recovering from COVID, showing that DTS [12] and repeated lung ultrasound [13] may represent valuable tools to track the evolution of COVID pneumonia. However, these imaging modalities can be of help mainly in patients demonstrating pulmonary interstitial changes or lung consolidations yet provide no information in terms of subtle functional alterations. In our study, chest quantitative CT analysis proved to be useful in identifying an overall reduction of lung volume occurring in patients who have recovered from COVID for several months, in the absence of detectable persistent lung changes.

Many patients do not recover completely after months following COVID acute infection. Townsend and coworkers demonstrated that a significant morbidity may persist after infection, affecting the perception of health, the ability to return to work, and the presence of enduring fatigue. This morbidity appears to be unrelated to the severity of the acute phase. This has implications for both the delivery of adequate health care to all patients with diagnosed COVID, irrespective of the need for hospitalization, as well as the economic impact on the workforce [24].

Recent research about the early convalescence phase suggests pulmonary function impairment as the most common sequela, reporting reduction in diffusing-capacity, FEV1/FVC ratio (Forced Expiratory Volume in the 1^st^ second/Forced Vital Capacity), DLCO abnormalities, obstructive or restrictive pulmonary dysfunction, and TLC decrease [3]. Usually, most of these patients did not show any particular pathological changes at Chest CT examination that resulted negative for macroscopic parenchymal findings.

Interestingly, our quantitative chest CT analysis showed a proportional lung volume reduction of both lungs in the post-COVID group. This finding may suggest a widespread diffusion of the virus throughout lungs and a global interstitial involvement despite the pattern of chest CT presentation that in most cases can be unilateral and more localized. In this setting, prolonged elevated levels of inflammatory serum markers (including IL-6, CRP, and TGF-β), which have been identified in patients at increased risk of developing pulmonary fibrosis after SARS-CoV-2 infection [25,26], may lead to diffuse microscopic fibrotic changes not detectable at standard chest CT imaging, with subsequent reduction in lung volume measurements.

During acute COVID infection, the importance of quantitative chest CT analysis of well-aerated lung expressed in both liters and percentage is well known in the literature due to its ability to predict for oxygen support, need for intubation, and patient death [27].

Several limitations should be considered in the present study. First of all, the use of two different CT scanners. A GE LightSpeed scanner was used to capture data on all non-COVID patients and 30% of post-COVID patients; a Siemens Somatom Force Dual Source Dual Energy chest CT was used to do 70% of post-COVID chest CTs. However, the only factors that mattered when evaluating the results of our investigation (scan time) were the same for both scanners (3–5 s) and the interval between the breath hold message and the beginning of the acquisition (approximately 6 s). Second, this study may have been enhanced by a connection with spirometry data. Our initial goal was to assess a potential loss in lung volume; we are now considering this element for a potential future advancement of the study. Third, the lack of data regarding chest CT or vaccination status of patients included in post-COVID group during the acute phase of the infection. In fact, the reduction in lung capacity seen in these patients may have been caused by prior vaccination or may have been a result of earlier COVID-related diseases such as GGO, consolidations, or interstitial thickening that later went away. The indications for chest CT in an acute context, which is limited to a certain patient type, made it almost hard for young patients to obtain these data. [28].

Our study brings us beyond appearances and beyond the classic radiological signs of chest CT, introducing a quantitative evaluation of lung volumes in patients recovering from COVID. To the best of our knowledge, our study is the first one to evaluate the importance of quantitative chest CT analysis in Long COVID patients. Indeed, a chest CT negative for inflammatory findings may induce clinicians to attribute Long COVID mild respiratory symptoms to anxiety, especially in young patients. However, in this setting, subtle diffuse fibrotic changes, not detectable at standard chest CT imaging, may persist, leading to a reduction in lung volume and subsequent functional respiratory complaints.

In conclusion, considering the continuous circulation of the virus among us for years to come, emerging long-term effects of SARS-CoV-2 infection may accumulate, strengthening the need to monitor COVID patients after their recover. It is difficult to establish to what extent these findings may contribute to the understating of Long COVID, and further exploration is surely needed, but this is another step to gain a wider knowledge of the potential long-term effects caused by this new virus.

## Figures and Tables

**Table 1:** Clinical and demographic characteristics of the patients included in the study.

Characteristic	All Participants (*n* = 154)	Post-COVID (*n* = 77)	Non-COVID (*n* = 77)	*p*-Value
Age, years	33 ± 5 (18–40)	33 ± 6 (18–40)	33 ± 5 (18–40)	0.999
Sex				
Male	76 (49%)	38 (49%)	38 (49%)	1.000
Female	78 (51%)	39 (51%)	39 (51%)	
Symptoms				
Cough	66 (43%)	28 (36%)	38 (49%)	0.262
Dyspnea	54 (35%)	25 (32%)	29 (38%)	
Chest pain	38 (25%)	18 (23%)	20 (34%)	
Fatigue	55 (36%)	33 (43%)	22 (29%)	
Factors affecting lung capacity				
Obesity	17 (11%)	8 (10%)	9 (12%)	0.952
Thoracic malformations	5 (3%)	2 (3%)	3 (4%)	
Smoking habit	39 (25%)	17 (22%)	22 (29%)	

**Table 2:** Comparison of lung volume quantification between the two groups.

Lung Volumes	Post-COVID (*n* = 77)	Non-COVID (*n* = 77)	*p*-Value
Mean Right Lung Volume	2.76 L (95% CI 2.16–3.36)	3.00 L (95% CI 2.43–3.57)	0.017
Mean Left Lung Volume	2.48 L (95% CI 1.94–3.02	2.72 L (95% CI 2.18–3.26)	0.010
Mean Total Lung Volume	5.25 L (95% CI 4.13–6.37	5.72 L (95% CI 4.62–6.82)	0.012

## Data Availability

The data that support the findings of this study are available on request from the corresponding author. The data are not publicly available due to privacy or ethical restrictions.
